# *In vitro* assessment of retention and resistance failure loads of complete coverage restorations made for anterior maxillary teeth restored with two different cast post and core designs

**DOI:** 10.4317/jced.55566

**Published:** 2019-03-01

**Authors:** Efpraxia Bakirtzoglou, Savvas N. Kamalakidis, Argirios L. Pissiotis, Konstantinos Michalakis

**Affiliations:** 1 Resident, Department of Prosthodontics, Aristotle University Faculty of Health Sciences, School of Dentistry, Thessaloniki, Greece; 2 Faculty, Department of Prosthodontics, Aristotle University Faculty of Health Sciences, School of Dentistry, Thessaloniki, Greece and Adjunct Assistant Professor, Division of Postgraduate Prosthodontics, Tufts University School of Dental Medicine, Boston, Mass; 3 Professor, Department of Prosthodontics, Aristotle University Faculty of Health Sciences, School of Dentistry, Thessaloniki, Greece; 4 Associate Professor and Director of Graduate Prosthodontics, Aristotle University Faculty of Health Sciences, School of Dentistry, Thessaloniki, Greece and Adjunct Associate Professor, Division of Postgraduate Prosthodontics, Tufts University School of Dental Medicine, Boston, Mass

## Abstract

**Background:**

The purpose of this *in vitro*study was to evaluate the retention and resistance form of complete coverage restorations supported by two different cast post and core designs.

**Material and Methods:**

Forty extracted maxillary central incisors were randomly divided into four groups of 10 specimens each (namely A, B, C and D). All specimens were endodontically treated and a uniform post space of 9mm was created. All prepared teeth had a 360o chamfer ferrule of 2mm in axial height measured 0.5mm coronally from the cementoenamel junction (CEJ) and an axial wall thickness of 1.5 mm. Specimens in groups A and C received cast post and cores with the standardized core design, where the core ended at the coronal part of the ferrule, while specimens in groups B and D received cores that were encircling the ferrule. Cemented complete coverage restorations in groups A and B underwent tensile load stress, while the restorations in groups C and D underwent compressive load stress until failure.

**Results:**

Teeth in group A exhibited a mean failure load of 326.14±83.67 N under tension, while teeth in group B exhibited a mean failure load of 332.79±80.38 N (*p*=0.858). Teeth in group C recorded a mean failure load of 1042.81±205.07 N, and in group D a mean failure load of 875.15±167.64 N (*p*=0.061) under compression was registered.

**Conclusions:**

The standard cast post and core design with a 2 mm of ferrule height offers superior resistance, although not statistically significant (*p*=0.061), when compared to the core design encircling the axial wall ferrule. Both cast post and core designs offer equal retention. However, different failure modes of decementation were noted.

** Key words:**Endodontically treated teeth, Post-and-core technique, Endodontic-post, Decementation, Root fracture, Ferrule effect.

## Introduction

The intended purpose of an endodontic post has always been to provide the retention needed for the core restoration (1). The post and core complex provides the foundation for the retention of the definitive restoration (2). The retention and resistance form of the core restoration should adhere to the basic principles necessary to support the complete coverage restoration (3). The survival rates of maxillary anterior teeth restored with post and core systems have been reported to be between 82.6-93% with follow-up periods of six to ten years (4,5).

Root fracture has been documented as the major complication of teeth restored with post and cores and complete coverage restorations (6). One of the detrimental causes leading to this outcome is attributed to the fact that anterior teeth are subjected to non-axial loads of 135o (7). Ideally, a post and core system should exhibit a fracture resistance which is higher than the average occlusal loads exhibited during function (2,8,9). Retention and resistance depend greatly on the length of the post (10,11). Adequate post length reduces the stresses exerted on the tooth and could also lead to better stress distribution (12). Dislodgement of the post was the most frequent complication reported on a 10-year retrospective study by Gomez-Polo *et al.* (5).

In a review study, Peroz *et al.* (13) have identified the following parameters, as being crucial for the restoration of endodontically treated teeth: (i) post length, (ii) post diameter, (iii) post design, (iv) post fixation, (v) post and core material, (vi) definitive restoration, and (vii) remaining coronal tooth structure. The post and core design parameter was taken as granted and was never evaluated, since it followed the tooth reduction principles for complete coverage restorations. It has been well established by numerous studies (5,14-17), that the importance of preserving 2 mm of coronal dentin height after preparation has a crucial role on the fracture resistance and prevention of root fracture of endodontically treated teeth. Various ferrule designs have been suggested (18-20), but currently there is little research to favor one design over the others (21,22). Although a plethora of post and core designs have been tested in the literature, only one study has focused on the core design itself (23). In this *in vitro* study the authors prepared the facial and palatal walls of the remaining tooth structure to create an external bevel of 30o to the long axis of the tooth, thus extending the core part of the post onto the coronal part of the tooth preparation. That specific design, if modified without the bevel preparation and extended to encircle the whole height of the tooth’s ferrule, might provide extra resistance to fracture for the endodontically treated teeth.

The purpose of the present *in vitro* study was to evaluate the retention and fracture resistance of complete coverage restorations supported by two different cast post and core designs. The null hypothesis was that there is no difference on the resistance and retention failure loads between the teeth restored with the two different cast post and core designs.

## Material and Methods

This study was conducted in accordance with the Declaration of Helsinki and was approved by the Institutional Review Board/Ethics Committee of the Aristotle University of Thessaloniki School of Dentistry (Protocol Number: 18/27-11-2015). Forty extracted maxillary human central incisors were obtained and were randomly divided into four groups of 10 specimens each ( Table 1). The overall mean root dimensions measured from the cementoenamel junction were 13.9±0.98 mm in length, 6.12±0.52 mm palato-lingually and 6.05±0.36 mm mesio-distally ( Table 2). All posts had a 9 mm length, while the mean length of apical gutta-percha was 4.9±0.98 mm. All teeth were vital with no carious lesions at the time of the extraction. The teeth were examined under a stereo microscope with x10 magnification (BH2, Olympus Corp., Tokyo, Japan) ‘to ensure the absence of surface fracture lines. Radiographic examination was also performed to eliminate the possibility of internal root resorption. Disinfection was implemented with a 5.25% hypochlorite solution for 1 hour after which the teeth were stored in an isotonic saline solution of 0.9% NaCl.

**Table 1:**
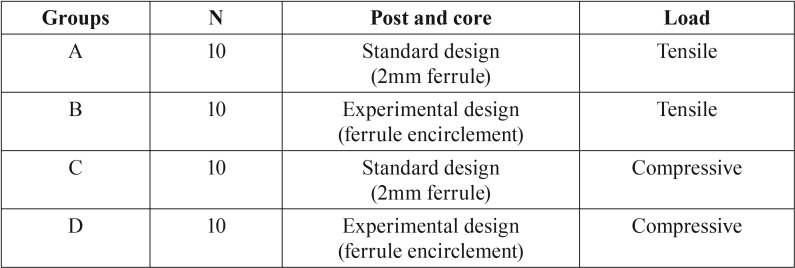
Distribution of specimens.

**Table 2:**
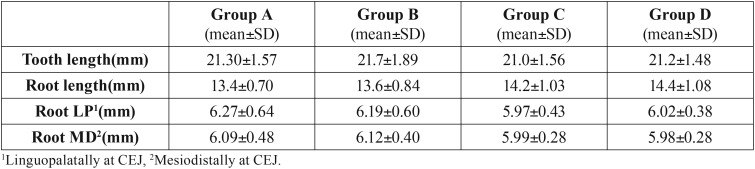
Mean specimen dimensions (n=10).

Access cavity preparation was initialized using a 2.1mm in diameter round diamond bur (#801-021C; SS White, Lakewood, NJ) and apical patency was verified with a size 15 K-file (Dentsply-Maillefer, Ballaigues, Switzerland). Endodontic instrumentation of the root canals was performed by using rotary Ni-Ti ProTaper files Sx-F4 (Dentsply-Maillefer, Ballaigues, Switzerland). The root canals were obturated using gutta-percha cones (Roeko; Coltene/Whaledent AG, Altstaetten, Switzerland) and AH-26 sealer (Dentsply-Maillefer, Ballaigues, Switzerland). The access cavities were sealed with a glass ionomer restorative material (Ketac-Molar; 3M/ESPE, St. Paul, MN) and the teeth were stored in 0.1% thymol solution for five days.

Following that period, each individual specimen of all four groups was positioned vertically by means of a surveyor (Ney Surveyor, Dentsply Inc., York, PA) at the center of a cylindrical plastic mold (55 mm in diameter), which was then poured with autopolymerizing polymethylmethacrylate (PMMA) acrylic resin (Vertex-Dental BV, Soesterberg, Netherlands) 2 mm below the CEJ. Each tooth specimen was mounted securely into a custom-made aluminum mold, which in turn was fixed to a multifunctional milling machine (BEGO, Bremen, Germany). All specimens were prepared equally, with a 360o ferrule design 2mm in height, measured 0.5mm coronally from the CEJ and an axial wall thickness of 1.5 mm. A silicone index key was fabricated to control the preparation depth of 1.0 mm with a uniform chamfer design at the finish line and a mesiodistal axial wall convergence of 6o. Teeth reduction was performed using medium and fine grit tapered diamond burs (FG857016; SS White, Lakewood, NJ), measuring 1.6 mm in diameter at the tip. Post spaces were prepared with Gates-Glidden drills #1-4 (Henry Schein, Inc., New York, USA), obtaining a uniform length of 9 mm for all specimens, measured from the most coronal part of the preparation.

**Figure 1:**
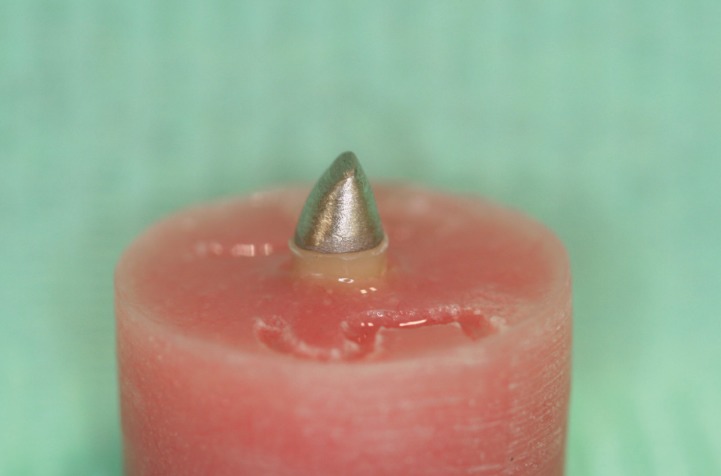
Experimental cast post and core design in groups B and D.

The post and cores were fabricated directly on the teeth using plastic burnout posts (Directa AB, Upplands Vasby, Sweden) and modelling and cervical wax (Thowax; Yeti Dental, Engen, Germany). Both the coronal part of the teeth and the root canals were lubricated with a non-oily, water soluble medium (Microfilm die lubricant; Kerr Dental, Orange, CA). In order to ensure uniformity of dimensions for the core part of the restorations, a silicone key was utilized, which was based on an initial pattern made out of autopolymerizing acrylic resin (Pattern resin LS; GC America, Alsip, IL). The height of the core part was set at 2 mm. The same procedure was repeated for both post and core designs of the study. Groups A and C utilized the standard core design, in which the core ended at the coronal part of the ferrule, while in groups B and D the cores were fabricated with the core ending at the internal finish line of the chamfer, thus encircling the ferrule (Fig. 1). The definitive post and cores were fabricated out of a silver-palladium alloy (Element P28; Element-Dental, Thessaloniki, Greece). In order to achieve undersized castings, the posts were cast without a ring liner (24). Finally, to achieve passive fit of the core portion in groups B and D, the technician applied two extra layers (four in total) of die spacer (Belle de st claire classic Blue; Kerr Corp., Orange, CA), providing 80μ of cement space (25).

Following the necessary laboratory procedures (i.e. casting, devesting, cleaning), the cast post and cores were air-abraded with 50-μm aluminum oxide particles under 2.8 kg/cm2 pressure and steam cleaned. Root canals were irrigated with distilled water and then dried using an air syringe and absorbent paper points. Then, luting was performed using a resin-modified glass ionomer cement (GC Fuji Plus; Tokyo, Japan) after tooth conditioning (GC Fuji Plus conditioner; Tokyo, Japan). The manufacturer’s instructions were followed for the luting procedure. Subsequently, 24 hours later, a single stage impression of all restorations was made by using a medium-body polyvinylsiloxane impression material (Virtual; Ivoclar-Vivadent, Schaan, Liechtenstein) and the definitive casts were obtained. Complete contour wax patterns were made for all four groups of the study. In groups A and B, a wax loop was incorporated at the coronal part of the wax pattern to assist in the tensile stress test. In groups C and D, a notch was designed at the palatal aspect of the patterns, 3 mm apically of the incisal edge to assist in the compression stress test. The wax patterns were invested with phosphate bonded investment (Fujivest II; GC America, Alsip, IL) and cast with a nickel chromium ceramic alloy (4all; Ivoclar-Vivadent, Schaan, Liechtenstein).

The completed restorations were cemented with the same luting agent used in the post and core cementation (GC Fuji Plus; Tokyo, Japan). Prior to the cementation the castings were air-abraded internally with 50-μm under 2 bars pressure and steam cleaned. All clinical steps of the study were performed by the same clinician, while the laboratory procedures were undertaken by the same experienced dental technician. The experimental procedures were performed at the Department of Basic Dental Sciences, Division of Dental Tissue Pathology and Therapeutics, School of Dentistry, Faculty of Health Sciences, Aristotle University of Thessaloniki, Greece. The cement was allowed to polymerize for 72 hours before the specimens were cleared for any testing procedure. Room temperature (21±2oC) and relative humidity (50±10%) were monitored throughout the study.

**Figure 2:**
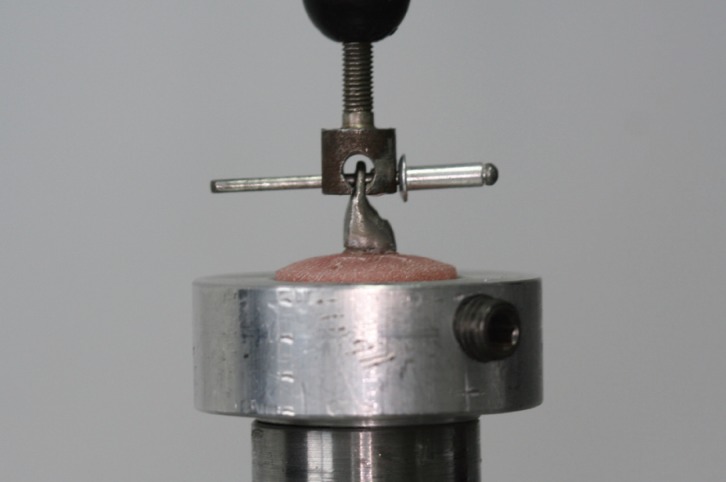
Specimen mounted on testing assembly for tensile load test.

**Figure 3:**
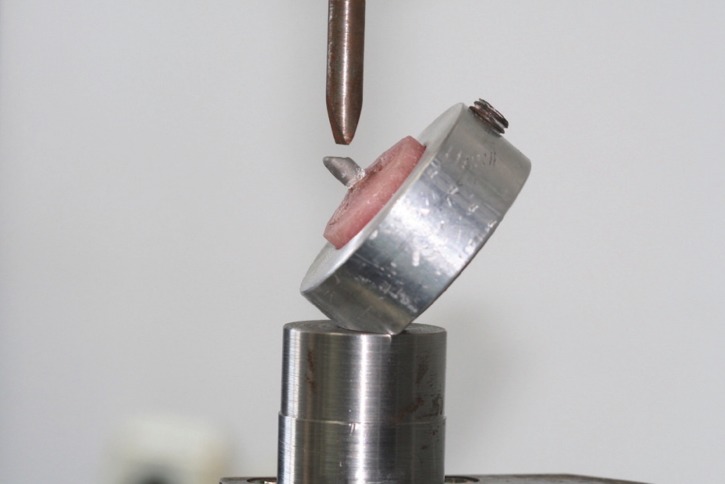
Specimen mounted on testing assembly for compressive load test.

Following the aforementioned period, each acrylic block was fixed inside a custom-made aluminum mold, which in turn was mounted in a universal testing machine (AX M350-10KN; Testometric Co Ltd, Rochdale, UK). Specimens in groups A and B, which were prepared for the tensile test, had a stainless-steel rod attached to the loop of the restoration (Fig. 2). The testing machine exerted a gradually increasing force parallel to the long axes of the teeth, until failure occurred. For the tensile stress test a load cell of 500N was used with a crosshead speed of 1.0 mm/min (26). A 10KN load cell was used for the compression test. A stainless-steel rod with a 3-mm-wide rounded end, applied the load to the palatal notch of the castings in groups C and D (Fig. 3), with a crosshead speed of 0.5 mm/min (27). The compressive load was exerted at a 135o angle to the long axes of the teeth until failure occurred. This angle was chosen as it represents an Angle’s Class I relationship. Maximum load values and modes of failure were recorded.

Descriptive statistics and the independent t test (a=.05) were used to determine the effect of tensile and compressive failure loads among the tested groups of the study.

## Results

The teeth in group A exhibited a maximum failure load of 326.14±83.67 N under tension, while the teeth in group B exhibited a maximum failure load of 332.79±80.38 N. Their difference was not found to be statistically significant (*p*=0.858). The difference under compressive failure load was also not statistically significant (*p*=0.061) between group C with a peak failure load of 1042.81±205.07 N and group D with a peak failure load of 875.15±167.64 N ( Table 3, Table 4).

**Table 3:**
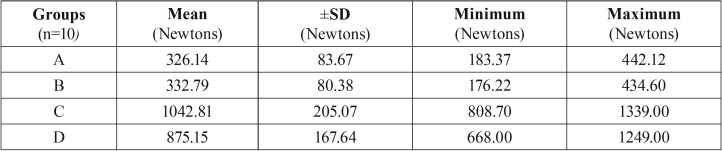
Peak force measurements for failure loads.

**Table 4:**
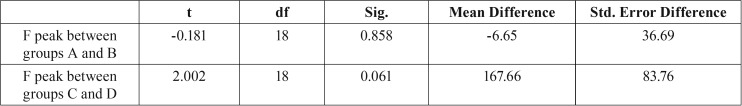
Independent sample t test for tensile and compressive failure loads.

The failure mode for the majority of the teeth in group A was decementation of the complete coverage restoration without any visible signs of root fracture, while for teeth in group B the prevalent failure mode was complete post decementation without noticeable root fractures. Teeth in groups C and D exhibited catastrophic root fractures, below the CEJ, in all cases.

## Discussion

The results of the present study confirmed the null hypothesis that both designs would behave equally under tensile and compressive loads. However, it should be pointed out that the standardized post and core design exhibited a higher failure load under compression compared to the experimental design with their difference resulting in a p value of 0.061. This finding could have been significant if a larger sample was utilized. Nevertheless, this needs to be verified with another study. Since this study’s experimental design was not utilized in any previous *in vitro* studies, no direct comparisons could be drawn. The mean failure load of the post and core in group C though, was comparable to the results of the study conducted by Shamseddine and Chaaban (23).

The failure mode of all teeth under compressive load was destructive root fracture. This has been observed in all cast post and core designs with adequate ferrule. The same finding was reported in previous studies, as well (2,14). It must be noted that the mean peak load before fracture of teeth in group D was considerably lower, although not statistically significant, than teeth of group C. This may be attributed to the fact that the encirclement of the remaining dentin band by the core portion of the cast post was possibly exerting additional stresses to the root. Further studies employing finite element analysis may be beneficial in verifying that assumption. A second possible source of stress could have been the tighter fit of the core around the ferrule. Since all post and cores in the study were cast without a ring liner to achieve an undersized casting as desired, the core portion around the ferrule needed extra attention to achieve passive fit of the post (25). All necessary procedures to verify the seating of the posts were observed throughout the study, but that could still have been a point of stress accumulation.

Furthermore, the failure mode of the majority of teeth (90%) under tensile stress was the full coverage restoration’s decementation in group A and post decementation in group B, with similar maximum failure loads. Since the teeth in all groups were of similar buccolingual and mesiodistal dimensions at the CEJ and all posts had an equal length of 9mm, the failure can be attributed to cementation surfaces. The full coverage restorations in group B had a greater surface area in contact with the core portion, which could explain the decementation of the posts, while the classic design of the cores in group A probably allowed the posts to remain intact. Adhesive failure was the common incidence reported in all similar *in vitro* and *in vivo* studies (2,6,12,13). It must be emphasized though that the restoration’s decementation could be more advantageous to the tooth’s future survival than the post’s decementation, as no bacteria will penetrate into the post space and recementation of the existing restoration could be readily performed.

A limitation of this *in vitro* study was that all specimens were subjected to a static loading, which does not accurately represent intraoral conditions. The loads exerted for either tensile or compression failure may have been smaller if a cyclic loading had been used. However, even cyclic loading cannot represent the oral environment as a standardized load is preset throughout the testing procedure. On the contrary, mastication must be considered as a rather complex procedure influenced by many parameters, such as gender, age, occlusal scheme, time, food texture, and the presence of temporomandibular disorders (7).

Future *in vitro* studies should test the influence of thermal cycling and fatigue loading on the retention and resistance form of different post and core designs. The results of the present *in vitro* study can only offer an indication as to the retention and resistance failure loads of the specific designs and should be confirmed by well-designed, long-term prospective clinical trials.

## Conclusions

Within the limitations of this *in vitro* study, the following conclusions can be drawn:

1. The classic cast post and core design with a 2mm ferrule design offers superior resistance, although not statistically significant, than the core design with the encirclement of the tooth ferrule.

2. Both designs offer equal retention, but with different failure modes of decementation (full coverage restoration vs post).
